# Cytokines and the neurodevelopmental basis of mental illness

**DOI:** 10.3389/fnins.2013.00180

**Published:** 2013-10-17

**Authors:** Udani Ratnayake, Tracey Quinn, David W. Walker, Hayley Dickinson

**Affiliations:** ^1^Ritchie Centre, Monash Institute of Medical Research, Monash UniversityClayton, Australia; ^2^Department of Obstetrics and Gynaecology, Monash UniversityMelbourne, VIC, Australia

**Keywords:** cytokines, mental illness, infection, pregnancy, stress, glial cells

## Abstract

Epidemiological studies suggest that prenatal exposure to different types of viral or bacterial infections may be associated with similar outcomes; i.e., an increased risk of mental illness disorders in the offspring. Infections arising from various causes have similar debilitating effects in later life, suggesting that the exact pathogen may not be the critical factor in determining the neurological and cognitive outcome in the offspring. Instead, it is thought that response of the innate immune system, specifically the increased production of inflammatory cytokines, may be the critical mediator in altering fetal brain development pre-disposing the offspring to mental illness disorders later in life. Inflammatory cytokines are essential for normal brain development. Factors such as the site of cytokine production, a change in balance between anti- and pro- inflammatory cytokines, placental transfer of cytokines, the effects of cytokines on glial cells, and the effects of glucocorticoids are important when evaluating the impact of maternal infection on fetal brain development. Although it is clear that cytokines are altered in the fetal brain following maternal infection, further evidence is required to determine if cytokines are the critical factor that alters the trajectory of brain development, subsequently leading to postnatal behavioral and neurological abnormalities.

## Introduction

Genetic background and environmental factors can predispose an individual to neuropsychiatric disorders, although the prevalence of conditions such as schizophrenia and autism cannot be accounted for by these factors alone (Williams et al., 2006; Tandon et al., 2008). The exact aetiology and pathophysiology of schizophrenia and autism remain unknown, but immunological factors are thought to play a role. Evidence of immune abnormalities including dysregulation of immune-related genes, changes in the activation of glial cells, and expression of inflammatory mediators have been observed in the brains of patients with these disorders (Pardo et al., 2005; Vargas et al., 2005; Arion et al., 2007; Chez et al., 2007; Morgan et al., 2010). It has therefore been suggested that immune-induced inflammation is a candidate event in creating a vulnerability in the brain that predisposes an individual, perhaps already at risk due to genetic and environmental influences, to develop the neuropathology and behavioral and cognitive impairments observed in major disorders such as schizophrenia and autism (Rehn and Rees, 2005; Meyer et al., 2011). Due to the debilitating nature of these conditions, their relatively high incidence in the community, and the great public health burden of these disorders, it is important to identify the causative factors in order to develop interventions and treatments for them, which might then become therapies that ameliorate or correct these disorders.

## Neurodevelopmental basis of mental illness

In the 1960s and 1970s, several studies showed an increase in the incidence of mental retardation in children born to mothers who were pregnant during the 1964 rubella epidemic (Stern et al., 1969; Chess, 1971; Chess et al., 1978). More recent studies, based on serological testing and clinical examination to determine rubella exposure during pregnancy have provided further convincing support for association between maternal infection during pregnancy and the development of mental retardation in the offspring (Brown et al., 2000a, 2001). The “1968 Hong Kong” influenza epidemic is another example of an exposure that, for women pregnant at the time, increased the risk of their children developing mental illness in later life—schizophrenia especially—with second trimester exposure being of particular significance (Mednick et al., 1988; Kendell and Kemp, 1989; Machón et al., 2002). In contrast, other studies concluded that prenatal exposure to influenza epidemics was unrelated to the development of mental illness in postnatal life (Crow and Done, 1992; Selten and Slaets, 1994; Morgan et al., 1997; Selten et al., 2009), but many of these were conducted retrospectively with influenza exposure based on timing of epidemics and a reliance on mothers to recall infection exposure after the pregnancy. These limitations weaken the validity of these studies and may be the reason an association between maternal influenza and an increased incidence of mental illness disorders was not found. In contrast, a study with convincing serological evidence to determine prenatal influenza exposure did find that influenza contracted during pregnancy increased the risk of schizophrenia in later life (Brown et al., 2004a).

Exposure to other infections in pregnancy have also been linked with the development of mental illness disorders, with an increased risk in children born to mothers where herpes simplex virus type 2 (Buka et al., 2001b) and toxoplasmosis (Brown et al., 2005; Mortensen et al., 2007) were detected during pregnancy. Epidemiological studies have also found that prenatal exposure to poliovirus (Fuller Torrey et al., 1988; Suvisaari et al., 1999), measles (Fuller Torrey et al., 1988), varicella-zoster (Fuller Torrey et al., 1988) and maternal genital and reproductive infections (Babulas et al., 2006) are all associated with an increased incidence of mental illness in the child in later life.

That similar outcomes can arise from quite varied infection in pregnancy suggests that responses in the maternal, placental or fetal compartments common to this type of immune challenge can result in altered development of the fetal brain. Importantly, whatever it is in the intra-uterine environment, or in placental and fetal tissues that is affected by maternal infection, they appear to induce long-lasting changes that, despite the much-discussed “plasticity” of the brain throughout life, nevertheless lead to a predisposition for the development of mental disorders in postnatal life, an outcome with debilitating consequences not only for the individual concerned, but also for their immediate family and society at a large.

## Are cytokines the critical mediator?

The numerous epidemiological studies showing an association between prenatal viral infections and an increased risk of mental illness in later life do not identify the critical factors in the disruption of fetal brain development. It is possible that factors common to the maternal immune system, and induced by any pathogen, viral or bacterial, may be affecting fetal brain development and leading to these postnatal behavioral disorders. Research has suggested that maternal induction or a change in the balance of pro- and anti-inflammatory cytokines may be a key mechanism involved in altering the normal course of fetal brain development (Gilmore and Jarskog, 1997; Ashdown et al., 2006; Meyer et al., 2009; Watanabe et al., 2010). It is important to note that other possible mechanisms by which a maternal infection could disrupt fetal development, include maternal and fetal malnutrition, hyperthermia, fetal growth restriction, and obstetric complications such as preeclampsia and preterm birth. As these factors do not consistently arise and are not common to all maternal infections, they are not discussed in depth in this review.

To eliminate microorganisms, such as bacteria and viruses, the innate immune system triggers the activation of Toll-like receptors (TLRs) (Takeda and Akira, 2005; Takeuchi and Akira, 2007). TLR activation triggers a cascade of signal transduction events causing the transcription of genes encoding cytokines, interferons and other immunological mediators (Takeda and Akira, 2005; Takeuchi and Akira, 2007). In the adult, cytokines, a family of soluble polypeptides, have been shown to be activated by these actions of the innate immune system, in response to both bacterial and viral infections (Imanishi, 2000; Borish and Steinke, 2003). It is thought that inflammatory cytokines not only play a key role in the resolution of disease, but in contrast, can also contribute to the development of inflammation (Hanada and Yoshimura, 2002).

Serological evidence further supports the concept that inflammatory mediators, produced in response to an infectious illness, may be involved in the development of mental disorders in postnatal life. Brown et al. (2004b) identified higher levels of serum interleukin (IL)-8 during pregnancy in mothers, particularly during the second trimester, whose child eventually developed schizophrenia. Increased serum levels of tumor necrosis factor (TNF)-α at birth have also been found in mothers whose child subsequently developed schizophrenia or other psychotic disorders (Buka et al., 2001a). Abnormalities in the constitutive expression of immunological factors also appear to be involved, in that chronically increased levels of IL-1β, IL-6, IL-8 and TNF-α are positively associated with the severity of the schizophrenia or autism that eventually develops (Maes et al., 2002; Chez et al., 2007; Potvin et al., 2008; Ashwood et al., 2011).

## The role of cytokines in normal brain development

Numerous studies have identified changes in cytokine levels throughout normal human pregnancy in maternal serum and amniotic fluid (Austgulen et al., 1994; Kruse et al., 2000; Heikkinen et al., 2001; Chow et al., 2008; Curry et al., 2008), but fewer have investigated cytokine levels in the human fetal brain through development, most likely due to difficulties in obtaining samples. A study conducted more than a decade ago, used *in situ* hybridization to find cytokine and chemokine expression in forebrain cells of human fetuses from 5 weeks of gestation (Mousa et al., 1999), suggesting a role for cytokines in normal brain development due to the presence of these factors from the first trimester. Animal studies have also demonstrated that cytokines are present in the fetal brain from early gestation (Meyer et al., 2006b, 2009) and have correlated increases in specific cytokines, such as TNF-α and IL-1β, with important development events in the brain (Dziegielewska et al., 2000). In terms of the human, the role of cytokines in normal brain development requires further research, particularly as this may be somewhat different between species. Differences may also exist in the function of specific cytokines in the prenatal and postnatal brain. For example, the cytokine IL-1β, is highly expressed in the fetal brain of rat and is thought to be an important astroglial growth factor during development (Giulian et al., 1988), but is expressed at a low level in the adult brain, although rapidly up-regulated as a host defence response to injury (Rothwell and Luheshi, 2000).

In the adult brain, cytokine expression occurs mostly in glia, predominantly microglia and astrocytes (Meeuwsen et al., 2003; Jack et al., 2005). A similar association has been found in the fetal brain, with studies *in vitro* having shown that cytokines and chemokines can be produced by human fetal microglia and astrocytes (Giulian et al., 1988; Hua and Lee, 2000; Lee et al., 2002; Rezaie et al., 2002a) and that cytokine production is increased by these glial cells in response to stimulation by a viral infection (Cheeran et al., 2001; Lokensgard et al., 2001). Similar to the adult brain, glial cells in fetal brain are also thought to receive and respond to signals from inflammatory cytokines (Hanisch, 2002). Furthermore, cytokines are thought to be critically important in glial cell development, in addition to neural and synaptic maturation (Deverman and Patterson, 2009).

## What is the source of the cytokines that induce fetal brain injury?

Many theories have been put forward to suggest how an infection during pregnancy could alter fetal brain development, and it is likely that cytokines play a major role. It is known that the activation of the maternal immune system by an infection during pregnancy induces the release of inflammatory cytokines (Shobokshi and Shaarawy, 2002; Zheng et al., 2012), but how these cytokines enter the fetal circulation and alter fetal brain development remains unclear.

Human and animals studies into the human influenza virus have shown that the virus or antibodies produced in response to the virus do not cross the placenta (Irving et al., 2000) and are not found in the brains of offspring where the mother had been exposed to influenza (Shi et al., 2005; Short et al., 2010; Fatemi et al., 2012). However, animal studies have shown that the activation of the maternal immune system by infections, such as influenza, alters cytokine levels in the placenta, amniotic fluid and fetal brain (Urakubo et al., 2001; Gilmore et al., 2005; Ashdown et al., 2006; Meyer et al., 2006b). These studies support the idea that it is not the virus that directly infects the fetal brain, but a substance released in either the maternal or placenta compartments that enters the fetal compartment and alters fetal brain development.

Studies have provided evidence that an increase in levels of cytokines in the maternal circulation could enter the fetal circulation, via the placenta, to influence fetal development. For example significant levels of cytokines were present in amniotic fluid and fetal tissues after the administration of specific cytokines, such as IL-2 and IL-6, to pregnant rodents (Dahlgren et al., 2006; Ponzio et al., 2007), indicating that these cytokines are able cross the placenta and enter the fetal circulation. In addition, the pro-inflammatory cytokine IL-6 was found to be capable of bi-directional transfer in a healthy term human placenta, although the same study also found that other cytokines including IL-1α and TNF-α show minimal transfer through the placenta and into the fetal circulation (Zaretsky et al., 2004).

As it still remains unclear how readily other cytokines and chemokines cross the placenta, the production of cytokines from the placenta itself is a second possibility to explain the presence of these substances in the amniotic fluid and fetal circulation. *In vitro* studies have shown that human trophoblast cells, from as early as the first trimester, respond to a viral mimetic that activates TLR-3 (Abrahams et al., 2006). As a result, type 1 interferons (IFN), inflammatory cytokines and other immunological mediators are inducted that may then enter both the fetal and maternal circulations.

Cytokines can be grouped according to their functions in the either inducing or suppressing inflammation. For example, IL-1β, IL-6, and TNF-α can be regarded as pro-inflammatory cytokines due to their role in early host defence against infection or disease, and in the development and progression of inflammation (Dinarello, 2000). Whereas cytokines such as IL-10 and transforming growth factor (TGF)-β can be considered as anti-inflammatory cytokines that have primarily immunomodulatory functions by limiting excess inflammatory reactions (Opal and DePalo, 2000). Researchers have hypothesized that it may not be the increased production and release of specific cytokines into the fetal compartment but instead a shift in excess pro- or anti-inflammatory cytokines during an infectious response, that may be the critical feature in disrupting normal brain development (Meyer et al., 2009; Patterson, 2009). For example, the over-expression of either the pro-inflammatory cytokine, IL-6 (Smith et al., 2007) or the anti-inflammatory cytokine, IL-10 (Meyer et al., 2008a) in pregnant rodents, causes similar cognitive and behavioral abnormalities in the offspring. As a consequence, it has been suggested that postnatal abnormalities would not arise if there were similar changes in both classes of cytokines, which therefore did not alter the balance between pro- and anti-inflammatory cytokines during fetal development (Meyer et al., 2009; Meyer, 2011). Although when a viral mimetic was administered to an IL-6 knockout mouse model or when co-administered with an IL-6 blocking antibody during pregnancy, the viral mimetic is no longer capable of producing the behavioral abnormalities and transcriptional changes in the offspring seen prior to IL-6 intervention (Smith et al., 2007). Instead this study suggests that the specificity of the cytokine response plays a key role in mediating the effects of a prenatal infection on the fetal brain.

Whether specific cytokines are derived in either the maternal or fetal system, or there is a change in the balance of cytokines, the stage of development of the fetal brain and/or immune system is an obvious factor in determining the consequences of a maternal infection. Epidemiological studies investigating various prenatal infections found that the risk of developing mental illness disorders can be dependent on the timing of exposure to an infection (Brown et al., 2000b, 2004a). In addition, studies investigating the administration of the viral mimetic, (Poly I:C) during gestation have provided further evidence that the time of prenatal exposure critically determines the patterns of behavioral and neurological abnormalities displayed in the offspring (Meyer et al., 2006a,b, 2008b). These studies found alterations in latent inhibition (Meyer et al., 2006a), exploratory activity (Meyer et al., 2006b) and sensorimotor gating (Meyer et al., 2008b), as well as deficits in an important neurodevelopmental market, Reelin expression (Meyer et al., 2006b) in the brain of offspring exposed to Poly I:C in early to mid gestation. Reversal learning deficits (Meyer et al., 2006b), impairments in spatial working memory (Meyer et al., 2008b) and increased apoptosis (Meyer et al., 2006b) were only demonstrated in mid-late prenatally exposed offspring. Cytokine levels in the maternal circulation and fetal brain also alter depending on the timing of Poly I:C administration, with greater levels of IL-10 and TNF-α when administered at early-mid compared to late gestation (Meyer et al., 2006a). These studies demonstrate that an immunological challenge at different times of prenatal development may have important, different neurodevelopmental consequences.

We propose two mechanisms by which an induction or a change in inflammatory cytokines during fetal life can have long term consequences on development; The effects of cytokines on 1) glial cells and 2) the hypothalamic-pituitary- adrenal axis.

## The effect of cytokines on glial cells

The suggestion that an increase of particular cytokines or a change in the critical balance between classes of cytokines caused by a prenatal infection may detrimentally alter normal fetal brain development, also assumes that glial cells are likely to be involved or at least affected as these cells are shown to not only produce cytokines but respond to them in the fetal brain (Hanisch, 2002).

Glial cells, such as astrocytes and microglia, are essential for normal brain development. Astrocytes are critical for the development of neurons and synapse formation during brain development and support neuronal repair and synapse formation (Benveniste, 1998; Ullian et al., 2004). In addition, astrocytes contribute to central nervous system homeostasis by supporting neuronal repair, contributing to the metabolism of neurotransmitters and regulating metabolite levels (Dong and Benveniste, 2001). For example, astrocytes are the major cell in the brain that produces kynurenic acid and quinolinic acid, N-methyl-D-aspartate (NMDA)-receptor antagonist and agonist, respectively, that modulate excitatory glutamatergic synapses. Therefore, an increase in astrogliosis also alters kynurenic metabolism, leading to changes in NMDA receptor activity (Schwarcz et al., 2012). Changes in the concentrations of these metabolites are associated with neurodegenerative and psychiatric diseases (Schwarcz et al., 2012), and it is now known that alteration of kynurenic acid synthesis during pregnancy in rats results in abnormal behavioral outcomes postnatally (Alexander et al., 2013; Forrest et al., 2013). In addition, the enzymatic activity of indoleamine 2,3-dioxygenase (IDO), which can be enhanced or inhibited by pro- or anti- inflammatory cytokines, respectively, can change the activation of the previously described kynurenic pathway (Myint et al., 2012).

Microglia are the resident immune cells of the central nervous system, and not only play a role in phagocytosis (Tremblay et al., 2011) but also produce cytokines and chemokines (Deverman and Patterson, 2009). In the adult brain resting microglia are thought to have a ramified morphology, with long branching processes that may be sensitive to (i.e., they “monitor”) the chemical composition of the cellular environment and identify the presence of injured cells and toxins associated with infection (Rezaie and Male, 1999). More recently researchers have suggested that “resting” microglia in the postnatal brain are dynamic in nature, continually extending and retracting these branching process (Nimmerjahn et al., 2005; Tremblay et al., 2011). In either case, ramified (or resting) microglia react to insult or injury by transforming into amoeboid microglia where the ramified processes are withdrawn and the central cell body region becomes enlarged (Bilbo and Schwarz, 2009; Deverman and Patterson, 2009). Amoeboid or activated microglia are actively phagocytic, and synthesize large amounts of inflammatory cytokines such as IL-1β, IL-6 and tumor necrosis factor (TNF)-α (reviewed in Bilbo and Schwarz, 2009). More recent studies (Rezaie et al., 2002b; Monier et al., 2007), utilizing technological advances in microscopy, have provided further confirmation of earlier findings (Kershman, 1939), that in contrast to the adult brain, fetal microglia display an amoeboid appearance when first present and progressively ramify during development.

As glial cells play an important role in normal brain development, it has been suggested that an activation or change in these cells, may alter the function of these cells and have a detrimental impact on the fetal brain (Perry et al., 2010). After exposure to a prenatal infection/inflammation, the predominantly amoeboid microglia in the fetal brain can potentially remain in this state into postnatal life (Bilbo and Schwarz, 2009; Hagberg et al., 2012), possibly exposing the fetal and postnatal brain to over-expression of pro-inflammatory cytokines, such as IL-1β, IL-6 and TNF-α [reviewed in Jonakait (1997); Bilbo and Schwarz (2009)].

As further evidence of the cytokine-glial hypothesis, alterations in structure and function of astrocytes and microglia have been identified in post-mortem studies of the brains of mental illness patients. Astrogliosis has been reported in human brain studies of autistic patients (Laurence and Fatemi, 2005). For schizophrenia this association is less clear, with different studies showing decreased (Webster et al., 2005), unchanged (Falkai et al., 1999) or increased (Arnold et al., 1996) levels of astrocytes in the brains of schizophrenic patients. Increased microglial cell number (Steiner et al., 2008), microglial activation (Bayer et al., 1999), and microglial cell density (Radewicz et al., 2000) in the post-mortem brains of schizophrenic patients are more consistent findings, as also for autistic patients (Morgan et al., 2010). The localization of activated (amoeboid) microglia has also been shown to be different in the prefrontal cortex, anterior cingulate cortex and hippocampal brain regions of schizophrenic patients (Steiner et al., 2006). As previously outlined, amoeboid or activated microglia produce large amounts of inflammatory cytokines including IL-1β, IL-6 and TNF-α [reviewed in Jonakait (1997); Bilbo and Schwarz (2009)]. Therefore, it is thought that the “priming” of microglia can cause an exaggerated cytokine response by these cells to a subsequent insult, compared to “unprimed” microglia (Czeh et al., 2011; Hagberg et al., 2012), thus exacerbating brain injury.

More recently, animal studies by us and others using prenatal immune activation, to cause behavioral neuropathologies comparable to mental illnesses encountered in the human population, have provided further evidence for the importance of microglial activation in pathogenesis of mental disorders such as schizophrenia. Microglial activation was found in brains of adult offspring following treatment of the mother with lipopolysaccharide during pregnancy (Borrell et al., 2002), and also in the brains of offspring following prenatal exposure to Poly I:C (Graciarena et al., 2010; Juckel et al., 2011; Ratnayake et al., 2012; Giovanoli et al., 2013). In such studies, conducted in pregnant rats and mice, the fetal brain is very immature at the time the infection raises an immune challenge and induces the cytokine changes described above. But even in a more precocial species such as the spiny mouse, where brain development is much more advanced by the time of birth, a very low dose (0.5 mg/kg) of prenatal Poly I:C treatment also increased microglial activation in the neonatal hippocampus (Figure 2) and led to persistent behavioral impairments in juvenile offspring (Ratnayake et al., 2012), confirming that prenatal subclinical infection has profound effects on brain development.

## The effects of cytokines on the hypothalamic-pituitary-adrenal axis

**Figure 1:**
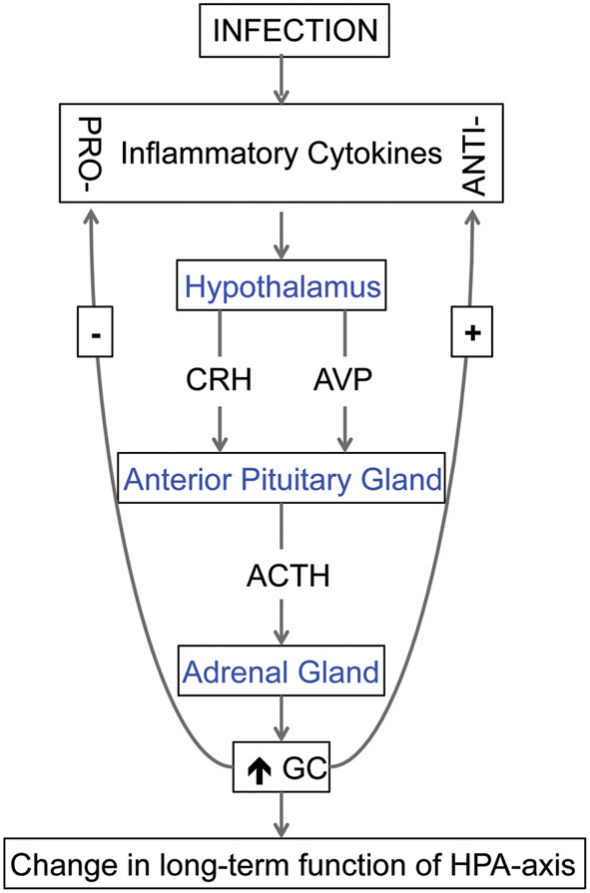
**The relationship between cytokines and hypothalamic-pituitary-adrenal (HPA) axis.** Cytokines, produced in response to an infection, can stimulate the release of corticotropin-releasing hormone (CRH) and arginine vasopressin (AVP) from the hypothalamus. These hormones stimulate the secretion of adrenocorticotrophic hormone (ACTH) from the anterior pituitary gland, and, in turn, the secretion of glucocorticoids (GC) from the adrenal cortex. During fetal life, excess GC can change the long-term function of the HPA-axis and if exposed to the fetal brain, GC can also modify brain development. In addition, GC can inhibit the induction of pro-inflammatory cytokines while stimulating the production of anti-inflammatory cytokines, a process that can also act to change the function of HPA-axis.

The immune system and the hypothalamic-pituitary-adrenal (HPA) axis, responsible for generalized stress responses, are inextricably linked (Franchimont et al., 2002; Chrousos and Kino, 2005; Silverman et al., 2005; Gibb et al., 2011). Cytokines can potently activate the HPA axis, to increase adrenocorticotropic hormone (ACTH), corticotropin-releasing hormone (CRH), arginine vasopressin and corticosterone levels (Besedovsky et al., 1986; Muller et al., 1999; Dunn, 2000; Schmidt et al., 2003). Therefore, cytokines, that have entered the fetal circulation due to a maternal infection, can potentially stimulate the release of CRH and arginine vasopressin from the fetal hypothalamus. The cytokine-elicited release of these hormones, shown in Figure 1, in turn, stimulates the secretion of ACTH from the anterior pituitary gland, and thus glucocorticoids from the adrenal cortex. In turn, glucocorticoids inhibit the induction of pro-inflammatory cytokines while stimulating the production of anti-inflammatory cytokines (Chrousos and Kino, 2005).

**Figure 2:**
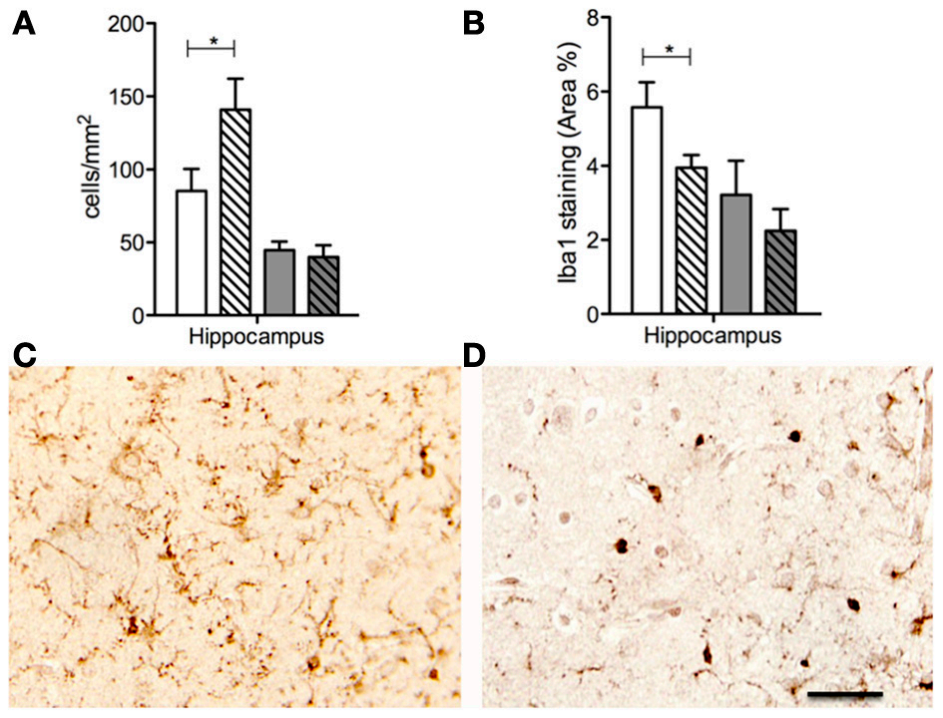
**Number of Iba1 immunopositive cell bodies (A) and percentage Iba1 staining in the hippocampus in PBS (solid bars) and Poly I:C (dashed bars) prenatally exposed animals at 1d (white bars) and 100d (gray bars) postnatal age.** Iba1 immunopositive staining in the hippocampus from animals prenatally exposed to PBS **(C)** and Poly I: **(D)** at 1d postnatal age. Note an increased number Iba1 immunopositive cell bodies in the Poly I:C **(A,D)** neonatal brains compared to the PBS **(C)**. Although, note an increase in the percentage of Iba1 staining in PBS neonatal brains **(B,C)** compared to Poly I:C **(D)**. The combination of these two findings, of more microglial cell bodies and fewer branching processes, make it possible to conclude that there are more activated, and fewer ramified, microglia in the neonatal hippocampus of animals born to mothers administered with the viral mimetic, Poly I:C. The data are shown as means ± SEM in all graphs. * refers *p* < 0.05. Scale bar = 100μM in **(C,D)**. Adapted from Ratnayake et al. (2012).

Although glucocorticoids are essential for normal brain development, exposure of the fetal brain to excess glucocorticoids can also modify fetal brain development and permanently alter the function of the HPA axis in postnatal life (Matthews, 2000; Welberg and Seckl, 2001; Seckl, 2004; Kapoor et al., 2006). Pro-inflammatory cytokines, such as IL-1, IL-6 and TNF-α, can alter the actions of glucocorticoids by interfering with their intracellular signaling system (Dunn, 2000; Bornstein et al., 2004; Giovanoli et al., 2013). The use of specific cytokine or glucocorticoid receptor knock-out mice in future studies could further help distinguish the role of each of these molecules during prenatal infection, and may reveal the link between infection and the neuroendocrine system that affects brain development.

The involvement of the HPA axis on cytokine changes in the fetal brain is dependent on the ability of the fetus to produce an appropriate stress response involving the HPA axis, and therefore, the time of the infection during pregnancy can be expected to modify the impact on the fetal brain development. Particularly as HPA axis development is strongly linked to the development of specific regions of the brain (Matthews, 2002). Hormones produced by the HPA axis can be detected early in development, by 12 weeks of gestation (Ng, 2000). The hormones, such as CRH and ACTH, have important actions during fetal development on the regulation of the HPA axis and the subsequent secretion of adrenal-derived glucocorticoids. Appropriate levels of glucocorticoids, such as cortisol and DHEA, are essential in maintaining intrauterine homeostasis and neuroprotective mechanisms, where as excessive levels of these hormones can be deleterious to fetal development.

It is well known that changes during fetal development can permanently programme the HPA axis, to have a long lasting impact on neuroendocrine function in postnatal life [reviewed in Matthews (2002); Bale et al. (2010); Howerton and Bale (2012)]. As it is not within the scope of this article to extensively review this concept, briefly, previous studies have demonstrated that adult animals exposed to a prenatal stressor, show DNA methylation and expression changes in glucocorticoid receptor and CRH (Nemeroff, 1992; Kapoor et al., 2008; Mueller and Bale, 2008). DNA modification by methylation can alter long term gene function, for example by silencing gene expression, without altering sequence variation. Therefore, it possible that a prenatal stress response, caused by exposure to an infection or inflammatory event, may result in the long lasting, epigenetic reprogramming of genes involved in HPA axis function.

## Conclusion

While mental illnesses such as schizophrenia and autism have been associated with prenatal infection, the variety of infections producing similar outcomes suggest that a response by the immune system common to all infections may be the important factor in the etiology of mental illness disorders. The common pathway may include induction of cytokines, triggered by activation of TLRs to assist in the resolution of disease but which then produce a pro-inflammatory environment that has downstream effects that are detrimental to fetal brain development. It is not entirely clear if significant amounts of cytokines enter the fetal circulation by placental transfer, or if placental and fetal production of cytokines is also important. However, the evidence is convincing that cytokines produced in response to infection during pregnancy have detrimental and long-lasting effects on the brain.

Inflammatory cytokines can affect the function of glia in the developing brain. Early deregulation of glial cells, such as microglia, may set in motion a cascade of events that lead to permanent alterations in these cells. There is substantial evidence for microglial activation in the post-mortem brains of patients that suffered a mental illness (Bayer et al., 1999; Radewicz et al., 2000; Steiner et al., 2006, 2008; Morgan et al., 2010), as well as in animal models of prenatal infection (Borrell et al., 2002; Graciarena et al., 2010; Juckel et al., 2011; Ratnayake et al., 2012), suggesting that permanently primed or “sensitized” microglia may be an important cellular component of the neuropathology of mental disorders that arise after birth. Another action of cytokines that may have long-term consequences is their ability to elicit a stress response via the activation of fetal HPA, which can subsequently cause long-term changes in HPA axis function in postnatal life.

It is possible to conclude that exposure to prenatal infection via the actions of cytokines can predispose or increase the susceptibility of an individual to future inflammatory events or postnatal stressors, as the function of the systems that act to resolve these insults have been permanently altered. Therefore, we believe it is important for future research to investigate two-hit models of mental illness disorders i.e., a first hit during fetal life—a prenatal viral or bacterial infection that drives an early susceptibility to mental illness via the activation of inflammatory pathways, such as the persistent activation of microglia, or changes in HPA axis function- in combination with a second hit during postnatal life—an immune activator or environmental factors such as chronic stressors or trauma. The development of these models may provide a unique period of time, prior to the “second hit,” to develop and target potential interventions or treatments to prevent the “full-blown” onset of a mental illness disorder.

### Conflict of interest statement

The authors declare that the research was conducted in the absence of any commercial or financial relationships that could be construed as a potential conflict of interest.
